# Mesenchymal stem cells with overexpression of midkine enhance cell survival and attenuate cardiac dysfunction in a rat model of myocardial infarction

**DOI:** 10.1186/scrt425

**Published:** 2014-03-17

**Authors:** Shu-Li Zhao, Yao-Jun Zhang, Ming-Hui Li, Xin-Lei Zhang, Shao-Liang Chen

**Affiliations:** 1 Department of Cardiology, Nanjing First Hospital, Nanjing Medical University, No. 68 Changle Road, Nanjing 210006, China; 2 State Key Laboratory of Reproductive Medicine, Nanjing Medical University, Nanjing, China; 3 Thoraxcenter, Erasmus Medical Center, Rotterdam, The Netherlands

## Abstract

**Introduction:**

Elevated midkine (MK) expression may contribute to ventricular remodeling and ameliorate cardiac dysfunction after myocardial infarction (MI). *Ex vivo* modification of signaling mechanisms in mesenchymal stem cells (MSCs) with MK overexpression may improve the efficacy of cell-based therapy. This study sought to assess the safety and efficacy of MSCs with MK overexpression transplantation in a rat model of MI.

**Methods:**

A pLenO-DCE vector lentivirus encoding MK was constructed and infected in MSCs. MSC migration activity and cytoprotection was examined in hypoxia-induced H9C2 cells using transwell insert *in vitro*. Rats were randomized into five groups: sham, MI plus injection of phosphate buffered saline (PBS), MSCs, MSCs-green fluorescent protein (MSCs-GFP) and MSCs-MK, respectively. Survival rates were compared among groups using log-rank test and left ventricular function was measured by echocardiography at baseline, *4*, *8* and *12* weeks.

**Results:**

Overexpression of MK partially prevented hypoxia-induced MSC apoptosis and exerted MSC cytoprotection to anoxia induced H9C2 cells. The underlying mechanisms may be associated with the increased mRNA and protein levels of vascular endothelial growth factor (VEGF), transformation growth factor-β (TGF-β), insulin-like growth factor 1 (IGF-1) and stromal cell-derived factor 1 (SDF-1a) in MSCs-MK compared with isolated MSCs and MSCs-GFP. Consistent with the qPCR results, the culture supernatant of MSCs-MK had more SDF-1a (*9.23* ng/ml), VEGF (*8.34* ng/ml) and TGF-β1 (*17.88* ng/ml) expression. *In vivo*, a greater proportion of cell survival was observed in the MSCs-MK group than in the MSCs-GFP group. Moreover, MSCs-MK administration was related to a significant improvement of cardiac function compared with other control groups at *12* weeks.

**Conclusions:**

Therapies employing MSCs with MK overexpression may represent an effective treatment for improving cardiac dysfunction and survival rate after MI.

## Introduction

Myocardial dysfunction after acute myocardial infarction (MI) is a progressive condition, which is clearly associated with a poor prognosis and results in heart failure and cardiac death [1-4]. Over the past decade, a number of studies have documented that mesenchymal stem cell (MSC) therapy may have a favorable impact on cardiac function in experimental animal models or patients after MI [5,6]. Our previous studies reported that autologous bone marrow MSCs transplantation improved cardiac function in *96* patients with acute MI who experienced percutaneous coronary intervention and the cardioprotective effect remained six months after the procedure [7,8]. Recently, studies from several preclinical experiments suggested that genetic strategies may play a critical role in improving MSC survival and differentiation [9-13]. However, insight into the mechanistic issues underlying the effect of genetically altered MSC transplantation remains unsettled, especially for finding a gene or a set of genes that potentially have both autocrine and paracrine effects in advancing MSC-directed myocardial repair.

Midkine (MK) is a heparin-binding growth factor with a molecular weight of *13* kDa, first isolated as a product of the retinoic acid-responsive gene in the embryonal carcinoma cell differentiation system [14]. MK has various biological activities, which promote neurite outgrowth, the survival of embryonic neurons, and angiogenic action [15]. In an experimental study comparing cardiac function after ischemia/perfusion (I/R) in wild-type mice and MK-deficient mice, there was a significant increased infarct area and adverse left ventricular (LV) fractional shortening (FS) in *MK−/−* mice [16]. Interestingly, supplemental application of MK protein to the *Mk−/−* mice at the time of I/R significantly reduced the infarcted size [16,17]. Alternatively, the studies from the H Takenaka and S Fukui groups showed that MK prevented the cardiac remodeling of mice after MI through an enhancement of angiogenesis and subsequently improved the survival rate. Apart from angiogenesis, additional research found that MK promoted the growth of mouse embryonic stem cells by inhibiting apoptosis through the PI3K/Akt signaling pathway [18]. Therefore, MK application is recently regarded as a new therapeutic strategy for the treatment of ischemic heart failure [19,20].

In the present study, we tested the hypothesis that the combination of MSC transplantation and MK overexpression is superior to MSC transplantation in the treatment of rat MI models with decreased infarct size and improved cardiac function. LV function and angiogenesis were separately evaluated by echocardiography and immunohistochemistry staining after transplantation. The biological activities of MSCs were also examined.

## Methods

### Animals

Healthy female Sprague-Dawley rats (weighing *60* to *80* g for isolation of MSCs and *200* to *220* g for an MI model) were obtained from the Vital River Laboratory Animal Co., Ltd., Beijing Laboratory Animal Research Center (Beijing, China); and were housed in specific pathogen-free conditions at Nanjing First Hospital Animal Center (Nanjing, China), in a room controlled for temperature (*21* ± *2*°C), humidity (*55* ± *5*%), and light (12-h light/dark cycle). Water was available to the rats *ad libitum*. After acclimation for two weeks, the rats were used for the study. All procedures involving animals were approved by the Ethics Committee for Animal Research of Nanjing Medical University.

### Isolation, culture and characterization of bone marrow mesenchymal stem cells

Bone marrow (BM) from the femur cavity was flushed using α-MEM medium (Invitrogen Corporation, Paisley, UK) containing 10% FCS (Hyclone Laboratories, Perbio Science, Cheshire, UK), 1% L-glutamine and 1% penicillin/streptomycin [21]. The cell suspension was centrifuged (*350* g, seven minutes), and cells were plated in culture flasks (*200,000* cells/cm^2^). Non-adherent cells were removed after *72* h. MSCs were recovered based on their capacity to strongly adhere to plastic culture dishes without cell sorting. MSCs were characterized by flow cytometry before recombinant lentiviral vector transfection using anti-rat CD90, CD29 FITC-conjugated antibodies and anti-rat CD44, CD34, CD31, CD86 PE-conjugated [22]. The antibodies and isotype-matched negative control antibodies labeled with FITC and PE were purchased from BD PharMingen (San Diego, California, USA) and used in all the experiments. Immediately before *in vivo* injection, the adherent MSCs and the genetically modified MSCs were detached with trypsin-EDTA, centrifuged for one minute at *1,200* *g*, and resuspended in PBS-BSA (*0.1%*).

### Construction of recombinant lentiviral vectors

A lentiviral vector system was selected because lentiviruses exhibit limited toxicity to infected cells. To construct the lentivirus encoding *MK* plasmid (pLenO-DCE-MK), the cDNA-encoding rat *MK* (NM_030859, 423-bp cDNA) was synthesized and cloned into the EcoRI and BamHI restriction endonuclease sites of the pLenO-DCE vector (cat. No. 26208-1, Invabio, Shanghai, China), a mammalian expression vector containing green fluorescent protein (GFP) and puromycin resistance genes. After the correct sequence was confirmed, lentiviral vector particles were produced in accordance with the manufacturer’s instructions (Invitrogen, Carlsbad, CA, USA). pRsv-REV, pMDlg-pRRE, pMD2G and pLenO-DCE-MK (or pLenO-DCE) were co-transfected into 293 T cells, and viral supernatants were harvested *48* and *72* hours after transfection, passed through 0.45-μm filters (Millipore corp., Bedford, Massachusetts, USA) and concentrated using four rounds of ultracentrifugation [23]. The viral pellet was resuspended in serum-free Dulbecco’s modified Eagle’s medium to obtain a 10,000-fold concentrate, and the debris was spun down. Viral stocks were stored at *-80*°C until use for transduction; functional viral titers were measured using QuickTiter Lentivirus Quantitation Kit (Cell Biolabs, San Diego, California, USA) and were determined by infection of 293 T cells [24].

### Transfection of cells with lentivirus encoding rat midkine

The MSCs were transfected with pLenO-DCE-MK vectors and pLenO-DCE control vectors as previously described [25]. Briefly, primary MSCs (*4 × 10*^*5*^ cells/well) were seeded in six-well plates (Costar, Corning, NY, USA) in complete culture medium. Twenty-four hours after seeding, MSCs were infected with recombinant lentivirus pLenO-DCE-MK vectors in multiples of *10, 20, 50* or *100* pfu/cell (MSCs-MK). The recombinant lentivirus encoding green fluorescent protein (pLenO-DCE) was used as a control (MSCs-GFP). The cells were incubated with the virus for at least *4* h in minimal culture medium, with shaking every *15* minutes. After *4* h of transfection, unbound virus was removed and replaced with fresh medium. The cells were incubated for another *48* h before treatment. After determination of the effect of infection, multiplicity of infection = *10* were chosen for the other experiment with sufficient overexpression of MK and minimum harm to the infected cells.

### Western blot analysis

MK overexpression was confirmed by Western blot analysis as previously described [26]. The MSCs, MSCs-GFP and MSCs-MK were lysed in lysis buffer (*50* mmol/L Tris–Cl, pH 8.0, *150* mmol/L NaCl, *0.02%* NaN3, *0.1%* SDS, *100* mg/L phenylmethylsulfonyl Xuoride, *1* mg/L aprotinin, and 1% Triton). Lysates were centrifuged at *12,000* rpm for *15* minutes. The supernatant was collected and denatured. Proteins were separated in *10%* SDS-PAGE gels and blotted onto polyvinylidene difluoride membranes (PVDF) (Merck Millipore, Darmstadt, Germany). The blot was blocked for *1.5* h at room temperature in *5%* BSA, followed by overnight incubation at *4*°C with anti-rat MK antibodies (Abcam, Cambridge, UK, clone # EP1143Y). Membranes were rinsed and incubated for *1* h with the corresponding peroxidase-conjugated secondary antibodies. MK protein was detected using an enhanced chemiluminescent reaction.

### Cytoprotective effects of MK overexpression to MSCs

To determine the cytoprotective effects of MK overexpression for MSCs, the oxygen and glucose deprivation (OGD) model was established *in vitro*[27]. After culturing for *24* h in six-well culture plates, the cell culture media from groups of MSCs, MSCs-DCE and MSCs-MK were replaced with glucose and serum-free Dulbecco’s modified Eagle’s medium (DMEM) (Gibco industries, Oklahoma, USA). The plates were then placed in a *37*°C anoxia chamber saturated with *95*% N_2_/*5%* CO_2_. At *12* h after incubation, apoptosis was analyzed using a flow cytometer to detect Annexin V-PE/7AAD staining (KeyGEN Biotech, Nanjing, China).

### Analysis of MSCs for growth factors and cytokines

To determine whether MK affected the paracrine factor secretions of MSCs, qPCR and ELISA were performed for the mRNA and protein levels of pro-angiogenesis factors (vascular endothelial growth factor (VEGF), transforming growth factor beta (TGF-β), fibroblast growth factor (FGF)2 and FGF7) and stem cell factors (stromal cell-derived factor (SDF)-1a, insulin-like growth factor (IGF)-1, granulocyte-macrophage colony-stimulating factor (GM-CSF) and stem cell factor (SCF)), respectively. Briefly, after culturing 1 × 10^6^/ml MSCs, MSCs-GFP and MSCs-MK cells for 48 h in six-well culture plates, the cells and the culture media were collected.

Total RNA was isolated directly from the MSCs, MSCs-GFP and MSCs-MK using TRIzol reagent (Invitrogen Life Technologies, Paisley, UK) and reverse-transcribed using a Super Script III reverse transcriptase kit (Invitrogen Life Technologies) according to standard protocols. Quantitative RT-PCR analysis was performed using a SYBR Green qPCR Master kit (Takara, Otsu, Shiga, Japan) and *300* mM primers (Table 1). After an initial *95*°C (*30* seconds) hot start, *40* cycles of *95*°C (*5* seconds) and *55*°C (*34* seconds) were performed using an ABI 7500 Real-Time PCR System (Life Technologies, NY, USA).

**Table 1:** The primers used for qPCR assays

**Genes**	**Forward**	**Reverse**	**cDNA size (bp)**
*VEGF*	CAGCTATTGCCGTCCAATTGA	CCAGGGCTTCATCATTGCA	131
*FGF2*	GGCTCTACTGCAAGAACGGC	GAAACAGTATGGCCTTCTGTC	353
*FGF7*	TTTGGAAAGAGCGACGACTT	GGCAGGATCCGTGTCAGTAT	209
*TGF-β*	TACAGGGCTTTCGCTTCAGT	TGGTTGTAGAGGGCAAGGAC	238
*SDF-1a*	TTTCACTCTCCGTCCACCTC	ATCTGAAGGGCACAGTTTGG	251
*SCF*	TTCGCTTGTAATTGGCTTTGC	TTCAACTGCCCTTGTAAGACTTGA	296
*IGF-1*	AAGCCTACAAAGTCAGCTCG	GGTCTTGTTTCCTGCACTTC	166
*GM-CSF*	GCTCACCCAACCCTGTCACCCG	CCTCATTTCTGGACCGGCTTCC	376
*β-actin*	AGGGAAATCGTGCGTGACAT	AACCGCTCATTGCCGATAGT^7^	149

The paracrine and secretion functions of MSCs, MSCs-GFP and MSCs-MK cells were evaluated by measuring the protein levels of IGF-1, SDF-1a, VEGF and TGF-β in their culture supernatants using the ELISA kits. After a 48-hour incubation of 1 × 10^6^/ml cells (fourth passage) in serum-free culture medium, the media was collected and centrifuged at *10,000* g at *4*°C for five minutes, and supernatants were stored at *-20*°C. Then, *100* μL of the supernatants were assayed for IGF-1 (Genway, Canada, GWB-ZZD062 sensitivity: <*62.5* pg/ml), VEGF (Abcam, UK ab100786, sensitivity: <*0.82* pg/ml), TGF-β1 (Abcam, UK ab119558, sensitivity: *31.3* pg/ml) and SDF-1a (Mybiosource, USA, MBS162802) using ELISA according to the manufacturer’s instructions.

### Cytoprotective effects of MSCs on H9C2 cells

The H9C2 cell line, an embryonic rat heart-derived cell line, was obtained from the Institute of Biochemistry and Cell Biology, the Chinese Academy of Sciences and maintained in DMEM supplemented with *100%* v/v fetal bovine serum and *100* mg/ml penicillin/streptomycin at *37*°C in a humidified atmosphere containing *5*% CO_2_. To induce hypoxia, 5 × 10^5^ H9C2 cells were planted in a six-well Transwell co-culture plate and placed in a *37*°C anoxia chamber saturated with *95*% N_2_/*5*% CO2 for eight hours and incubated with serum free and glucose free DMEM as described above.

To observe the impact of MK on MSCs migration and cytoprotection, MSCs-GFP and MSCs-MK were collected and seeded in the top well of a Transwell insert (Millipore, USA) at a density of *2 × 10*^*5*^ cells/well in *400* μL DMEM containing 10% FCS. For inhibition experiments, all MSCs were pre-incubated with CXCR4 antagonist AMD3100 (*10* μg/mL, Sigma-Aldrich, Shanghai, China), the VEGFR inhibitors Sorafenib (*20* nM, Selleck Chemicals, Houston, Texas, USA) and the same concentration vehicle control for *30* minutes before seeding.

The hypoxic H9C2 cells were added to the bottom wells of the Transwell plates. DMEM containing *10%* FCS was used as a migration control. After being cultured at 37°C in a humidified atmosphere of *5%* CO_2_ for *12* h, the cells in the bottom wells were collected, the proportion of GFP(+) cells was determined to evaluate the MSC migration activity, the apoptosis ratio was measured with a flow cytometer using Annexin V-PE/7AAD staining and the caspase-3 activity was measured by Western blotting. Each assay was carried out in triplicate.

### Myocardial infarction model

All animals received humane care in compliance with the ‘Guide for the Care and Use of Laboratory Animals’ prepared by the Institute of Laboratory Animal Resources, the National Research Council, and published as the ‘Guide to the Care and Use of Experimental Animals’ by the Chinese Council on Animal Care. All procedures involving animals were approved by the Ethics Committee for Animal Research of Nanjing Medical University. MI was induced in *70* experimental rats by ligating the left anterior descending (LAD) coronary artery as previously described [28], with some modifications. Briefly, rats were anesthetized with sodium pentobarbital (*50* mg/kg intraperitoneally) and intubated and ventilated. A left lateral thoracotomy in the fourth intercostal space was performed to expose the anterior surface of the heart. The proximal LAD coronary artery was ligated with a 6.0 polypropylene snare (Ethicon Inc., Somerville, New Jersey, USA). The area displaying tissue blanching and wall motion akinesis was identified as the infarct. Rats in the sham group underwent the same procedure except for ligation of LAD.

### Implantation of MSCs

Seventy rats were used to establish the infarcted model. Sixty rats were selected from the *62* surviving ligated animals, *15* of which were randomly re-selected as the model-assessment group for baseline evaluation of the heart infarcted size. Two weeks after the ligation, the *60* model rats were equally randomized to one of four groups: (1) the MSCs group (n = *15*), in which MSCs in suspension were injected intramuscularly at the left anterior free wall using a 30-gauge needle; (2) the MSCs-GFP group (n = *15*), in which the animals were injected intramuscularly with pLenO-DCE transfected MSCs suspension; (3) the MSCs-MK group (n =* 15*), in which the animals were injected intramuscularly with pLenO-DCE-MK transfected MSCs suspension; and (4) the PBS group (n = *15*), where the animals were injected with PBS. PBS or cell solutions were injected at three injection sites into anterior and lateral aspects of the viable myocardium bordering the infarction (total *5.0 × 10*^*6*^ cells in *0.1* mL). After injection, the chest was closed and the animals were allowed to recover.

### Echocardiography measurements

Echocardiography measurements in a blinded fashion were performed one day before MI induction (baseline) and *4, 8* and *12* weeks after implantation in anesthetized rats (*2%* isoflurane inhalation) using a Vevo 770 cardiac system (VisualSonics Inc., Toronto, ON, Canada). Left ventricular end-diastolic diameter (LVEDD), LV end-systolic diameter (LVESD), and LVFS were recorded from the parasternal long-axis M-mode images using averaged measurements from three to five consecutive cardiac cycles in accordance with the American Society of Echocardiography guidelines. Left ventricular end-diastolic and end-systolic volumes (LVEDV and LVESV) were calculated from bidimensional long-axis parasternal views taken through the infarcted area by means of the single-plane area-length method [17]. The LV ejection fraction (LVEF) was calculated as follows: LVEF = (LVEDV-LVESV)/LVEDV) × 100%.

### Histological analysis

We performed PCR to further confirm the promotion by quantifying the mRNA expression of GFP in the LV free wall of rat hearts. The primers of 5′-GAGCTGAAGGGCATCGACTT-3′ and 5′-CTTGTGCCCCAGGATGTTG-3′ were used in PCR amplification to detect GFP in MSCs-GFP and MSCs-MK.

Capillary density was determined by immunohistochemical staining with anti-CD31 antibody after cell therapy, as previously described. The tissue sections (5 μm) were stained for CD31 (Santa Cruz Biotechnology Inc., TX, USA) to identify capillaries. Immunohistochemical staining was performed using a two-step immunohistochemical technique with DAB (Maixin Bio, Fuzhou, China), as described in the manufacturer’s specifications. After being restained with hematine, the samples were cover-slipped and photographed. The cytoplasm of the endothelial cells was stained red. The capillaries were counted with a × 200 microscopic objective in 10 randomly selected fields in two sections per animal and averaged. The criteria for being counted consisted of having diameters of less than 50 μm and including single or tiny vascular endothelial cells.

To detect fibrosis and apoptosis in cardiac muscle, the hearts were excised, cut transversely, embedded in paraffin, stained with Masson’s trichrome and the TUNEL kit (Boster Bio, Wuhan, China), photographed and analyzed. The blue area was regarded as fibrotic tissue, and the brown area was regarded as apoptosis tissue.

### Statistical analysis

Data are presented as the mean ± standard deviation. Statistical analysis was performed using Student’s *t*-test or ANOVA, as appropriate. Differences in echo parameter changes from baseline for each measured variable were assessed with repeated-measures, one-way analysis of variance (ANOVA) followed by Dunnett’s multiple-range test to evaluate changes of the measured variable from the corresponding baseline within the same animal. Differences between groups in echo parameters were determined by two-way ANOVA followed by Bonferroni multiple comparison post-tests when the ANOVA indicated significant differences in echo parameters. *P* <0.05 was considered statistically significant. All statistical analyses were performed using SPSS 20.0 (IBM Corp., Armonk, NY, USA).

## Results

### Characterization of MSCs-MK

**Figure 1:**
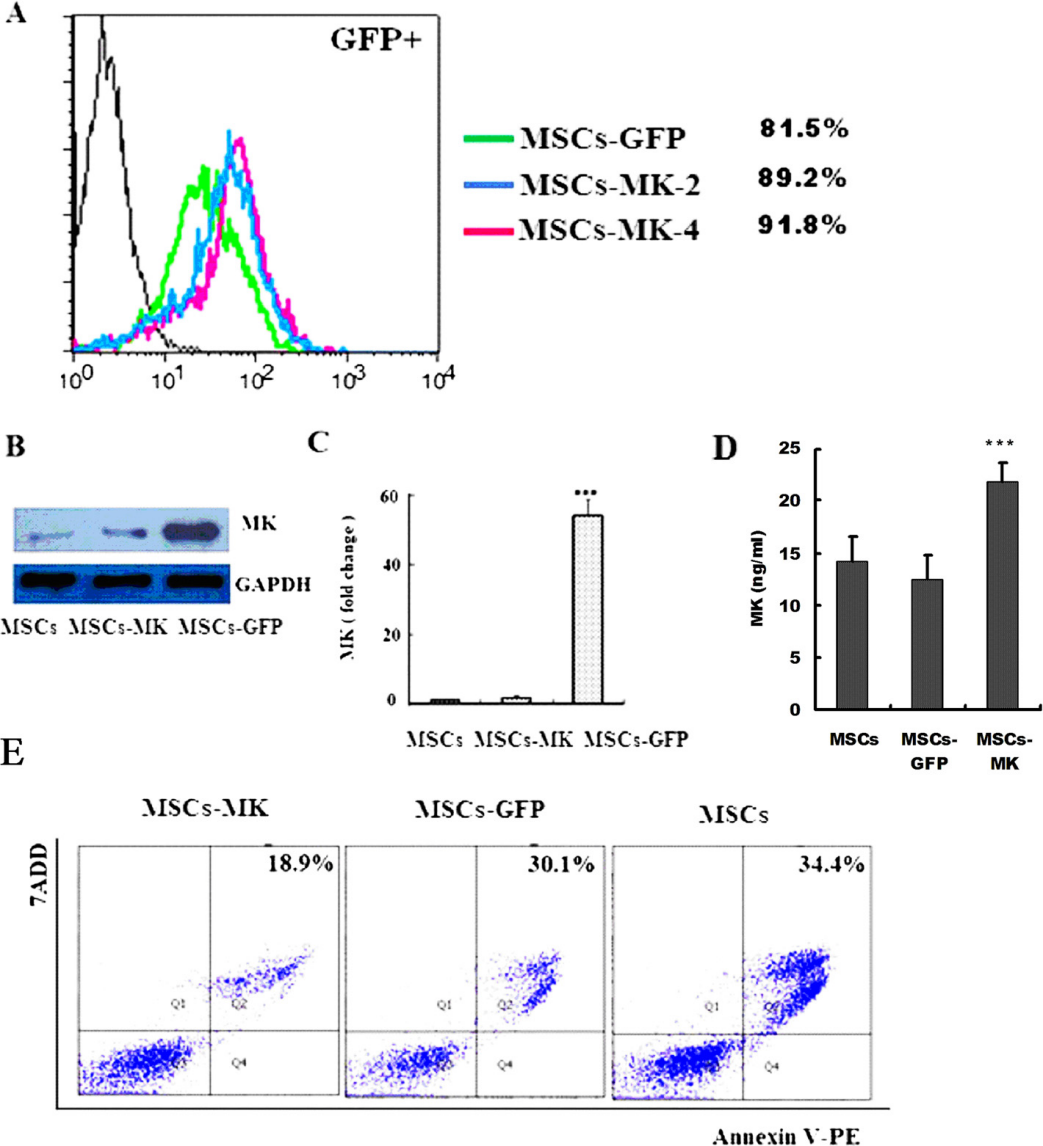
**Characterization and cytoprotective effects against anoxia of MK-transduced mesenchymal stem cells. (A)** Flow cytometry analysis of the efficiency of lentivirus affection. Black: MSCs, green: MSCs-GFP, blue: MSCs-MK two generations after lentivirus infection, red: MSCs-MK four generations after lentivirus infection. **(B)** Quantitative real-time polymerase chain reaction of MK expression. **(C)** Western blot of MK protein levels. **(D)** ELISA of MK protein levels in the culture supernatants of MSCs, MSCs-GFP and MSCs-MK. **(E)** Flow cytometry analysis of cell apoptosis in all groups. MSC, mesenchymal stem cell; MSCs-MK, MSCs overexpressing midkine. The data are shown as the means ± SEM of three independent experiments. ***p< 0.01 versus control. MSCs-GFP, Mesenchymal stem cells-green fluorescent protein; MSCs-MK, Mesenchymal stem cells-midkine.

After three passages, the adherent MSC cells were symmetric with phenotypic surface antigens as reported, including positivity for CD29, CD44 and CD90 and negativity for CD34, CD31 and CD86 (>*90%*, Figure S1 in Additional file 1) [22,29,30]. We successfully developed the high-titer lentiviral vectors that drive expression of rat MK/GFP (pLenO-DCE-MK, *1.5 × 10*^*9*^ TU/ml) and GFP (pLenO-DCE, *2.0 × 10*^*9*^ TU/ml) (Figures S2 and S3, Additional file 1). After infection with recombinant lentivirus, the efficiency of the gene transduction of MSCs to overexpress MK was similar to that of mock lentivirus *(95.2 vs. 95.3%)* (Figure 1A); moreover, above six phenotypic surface antigens did not change (data not shown).

### Overexpression of MK improved MSCs survival

Lentivirus-mediated transduction and expression of MK were confirmed with real-time PCR and Western blotting. Quantitative real-time PCR data indicated that expression of MK was 54.2-fold higher in MSCs-MK (Figure 1B). MSCs-MK also exhibited higher levels of MK protein (Figure 1C); moreover, the midkine was secreted (Figure 1D). To investigate the resistance of MSCs-MK to anoxia, MSCs, MSCs-GFP and MSCs-MK were exposed to oxygen and glucose deprivation and analyzed with the Annexin V-PE/7ADD kit. MSCs displayed morphological changes of apoptosis and necrosis after anoxia. Overexpression of MK, however, partially prevented apoptosis induced by anoxia. The numbers of apoptotic and necrotic cells *(17.9 ± 4.2%)* decreased significantly in the MSCs-MK group compared with the MSCs and MSCs-GFP groups (*28.4 ± 3.7%, p< 0.05; 32.6 ± 4.9%, p< 0.05;* Figure 1E).

### MK enhanced the paracrine effects of MSCs

**Figure 2:**
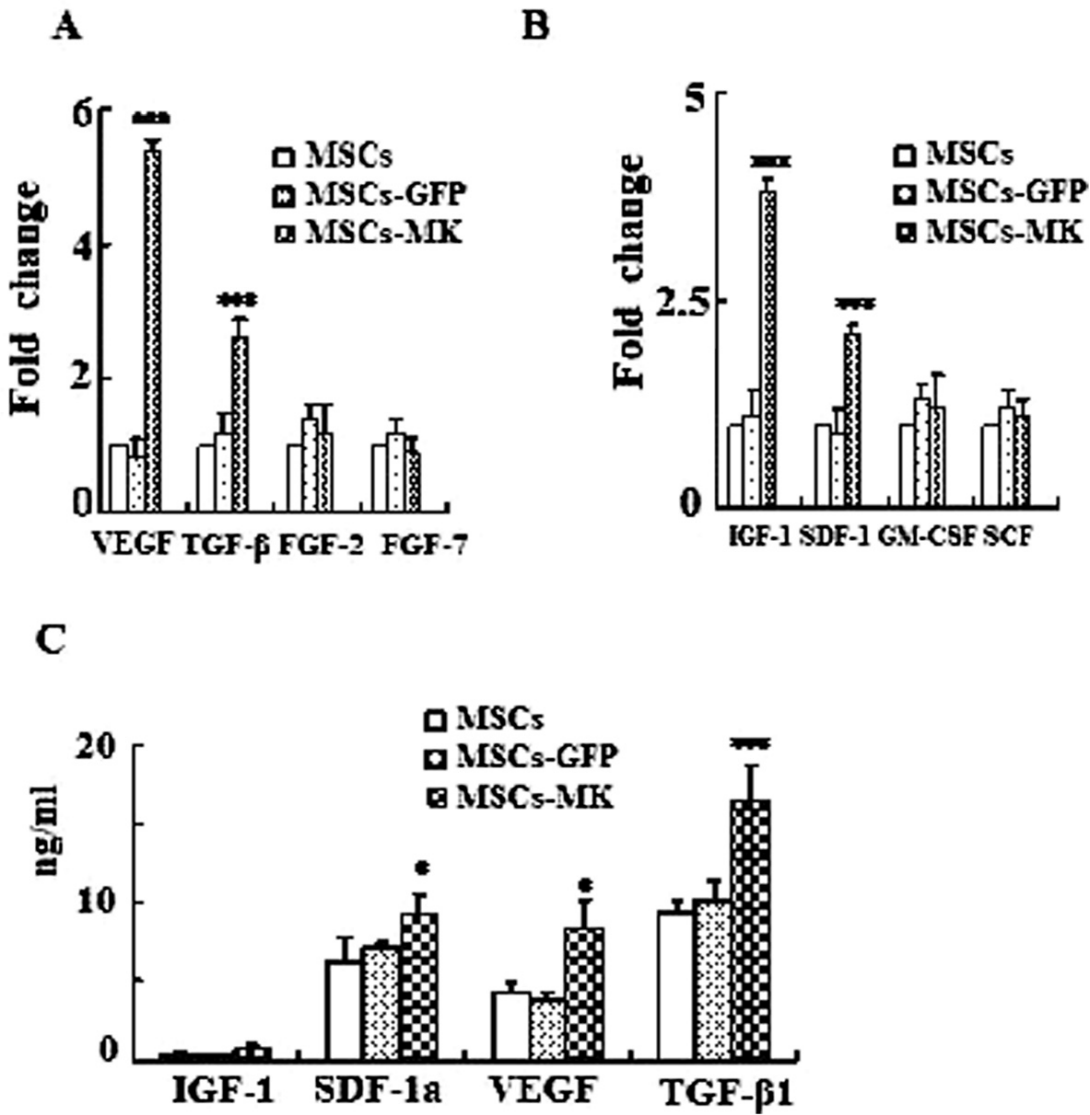
**MK increases the paracrine effects of MSCs. A** and **B**: Real-time PCR analysis of pro-angiogenesis factors (VEGF, TGF-β, FGF2 and FGF7) and stem cell factor (SDF-1, IGF-1, GM-CSF and SCF) mRNAs. The relative expression of VEGF (5.85-fold), TGF-β (2.78-fold), IGF-1 (3.7-fold) and SDF-1 (2.01-fold) mRNA in MSCs-MK were higher than in control cells (***p<0.01). **C**: ELISA of IGF-1, SDF-1a, VEGF and TGF-β1 protein levels in the culture supernatants of MSCs, MSCs-GFP and MSCs-MK. MSCs-MK secreted more SDF-1a (9.23 ng/ml), VEGF (8.34 ng/ml) and TGF-β1 (17.88 ng/ml) than MSCs (6.35, 4.75 and 9.18 ng/ml) and MSCs-GFP (6.92, 4.55 and 9.88 ng/ml); this was not the case for IGF-1 (MSCs-MK, 1.89 vs. MSCs, 1.51 and MSCs-GFP 1.40 ng/ml). FGF, fibroblast growth factor; GM-CSF, Granulocyte-macrophage colony-stimulating factor; IGF-1, Insulin-like growth factor 1; MSCs-GFP, Mesenchymal stem cells-green fluorescent protein; MSCs-MK, Mesenchymal stem cells-midkine; SCF, Stem cell factor; SDF-1, Stromal cell-derived factor 1; TGF-β, Transforming growth factor beta; VEGF, Vascular endothelial growth factor.

It is known that the main mechanisms by which MSCs can increase repair on myocardial infarction are cardiogenic differentiation (direct contribution), autocrine effects (affecting the MSCs themselves) and paracrine effects (affecting the wounded/infracted tissue) [31]. In the present study, we did not find any evidence that MK could promote MSC differentiation into cardiomyocytes (data not shown). To determine whether MK impacts the expression of pro-angiogenesis and some stem cell-related factors, we measured mRNA of VEGF, TGF-β, FGF2, FGF7, SDF-1a, IGF-1, GM-CSF and SCF in MSCs, MSCs-MK and MSCs-GFP respectively (Figure 2A,B), and the protein levels of IGF-1, SDF-1a, VEGF and TGF-β1 were also quantified by ELISA (Figure 2C). The mRNA levels of VEGF, TGF-β, IGF-1 and SDF-1a were elevated *5.85-, 2.78-, 3.7-* and *2.01-*fold, but no elevation in the mRNA levels of FGF-2, FGF-7, GM-CSF and SCF was observed in MSCs-MK compared with MSCs and MSCs-GFP, indicating that this elevation was specific for MK overexpression. Consistent with the qPCR results, MSCs-MK secreted more SDF-1a (*9.23* ng/ml), VEGF (*8.34* ng/ml) and TGF-β1 (*17.88* ng/ml) than MSCs and MSCs-GFP; this was not the case for IGF-1 (*1.89* ng/ml).

### MK increased the protective effects of MSCs on cardiomyocytes

**Figure 3:**
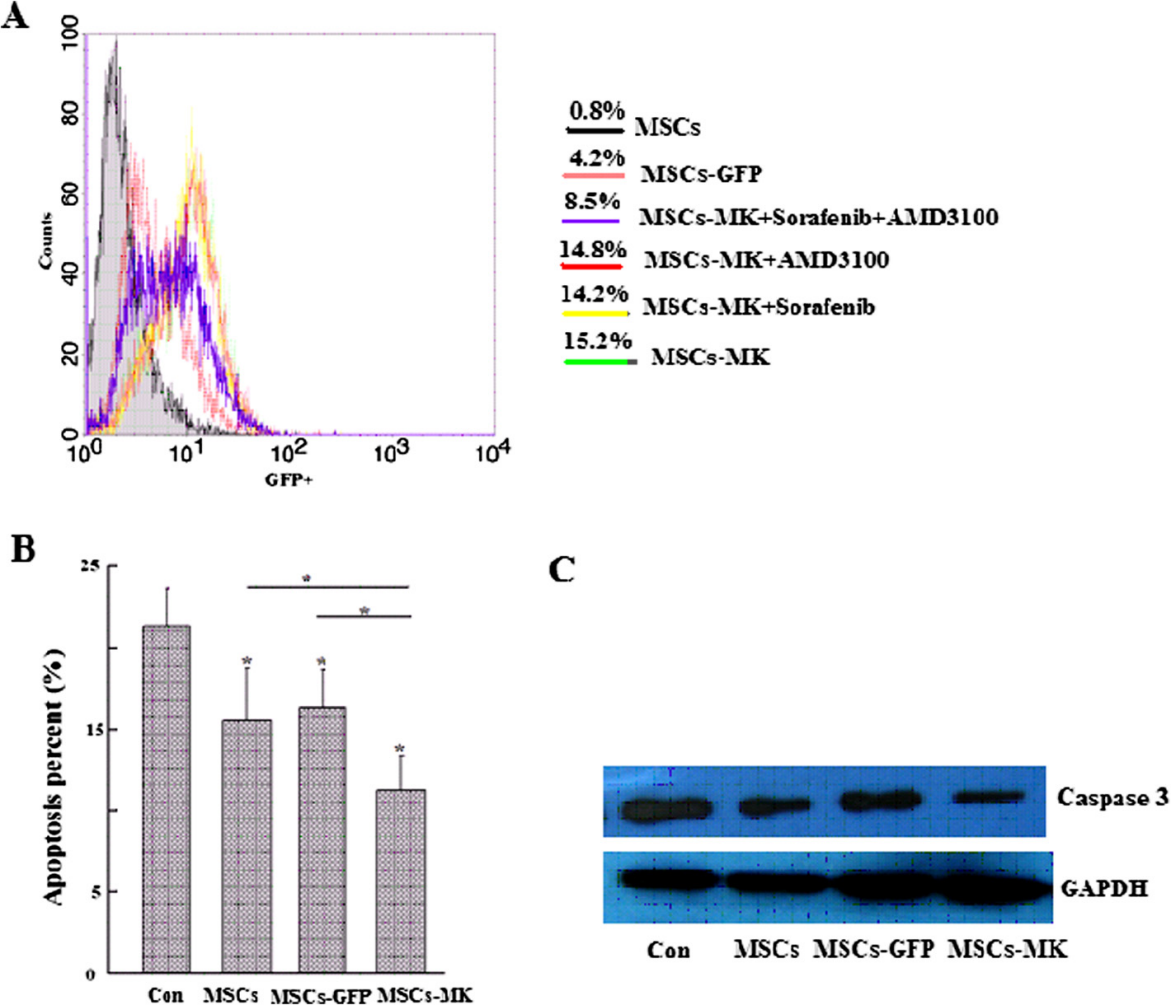
**MK increased the protective effects of MSCs on H9C2 cells. A**: Flow cytometry analysis of the proportion of GFP + cells in the bottom hypoxic H9C2 wells. **B**: Protective effects of MSCs, MSCs-GFP and MSCs-MK on hypoxic H9C2 cells, measured by Annexin V-PE/7AAD staining. The graph shows the percentages of Annexin V-PE + and 7AAD + (apoptotic) cells as the means ± SEM for three independent experiments. **C**: Caspase-3 activity of H9C2 was analyzed by Western blot. MSCs-GFP, Mesenchymal stem cells-green fluorescent protein; MSCs-MK, Mesenchymal stem cells-midkine.

The secreted factors of MSCs exert biological activities by binding to their corresponding receptors on cardiac cells. To determine the ability of MSC homing to the site of injury and the protective effects of MSCs-MK on cardiomyocyte viability in an *ex vivo* model of hypoxia, the hypoxic H9C2 cells were co-cultured with MSCs, MSCs-MK and MSCs-GFP in six-well Transwell plates. Flow cytometric analysis showed that there were more GFP^+^ cells in the bottom H9C2 wells in the MSCs-MK group than the MSCs-GFP group (Figure 3A). As shown in Figure 3A, the migratory response of MSCs-MK to H9C2 was increased two-fold compared with MSCs-GFP. Although both the VEGFR inhibitors (Sorafenib, Selleck, USA) and the CXCR4 antagonist (AMD3100) could not inhibit MSCs-MK migration, the increased migration was blocked by a combination treatment (Figure 3A), suggesting that MK enhanced MSC migration at least partially through the VEGF/VEGFR and SDF-1a/CXCR4 pathways.

H9C2 myoblasts were used to test the hypothesis that paracrine factors secreted by MSCs inhibit the H9C2 cell apoptosis induced by anoxia. The hypoxic H9C2 cells were co-cultured with MSCs, MSCs-GFP and MSCs-MK in six-well Transwell plates, and then, the apoptosis rate and caspase-3 activity of H9C2 cells were analyzed. As shown in Figure 3B,C, the apoptosis ratio and caspase-3 activity of H9C2 cells were significantly reduced in the MSCs-MK group compared with the other two groups (*p< 0.05*).

### Injection of MSCs-MK prevented cardiac dysfunction after MI

**Figure 4:**
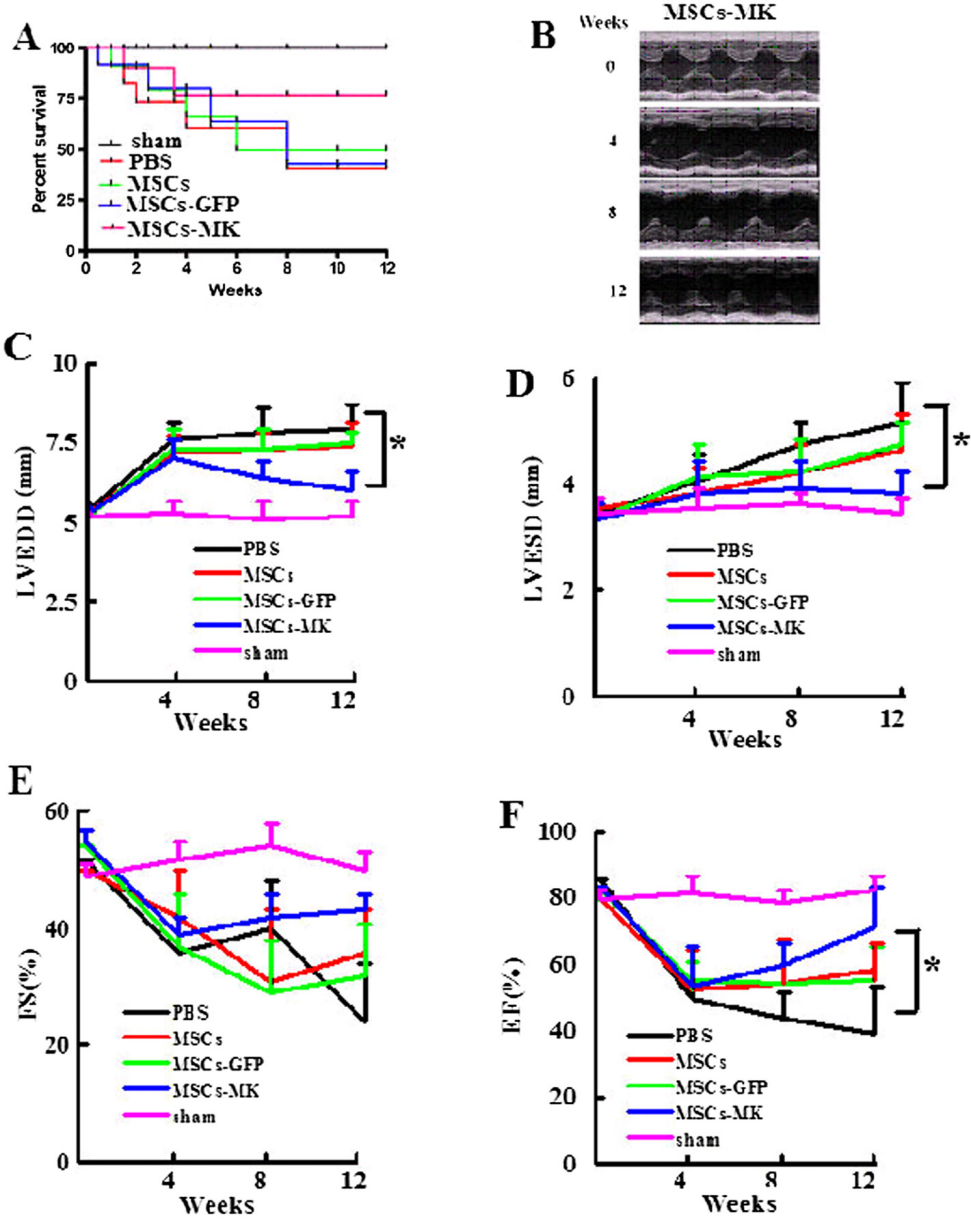
**Curative effect evaluation of MSCs-MK by echocardiography in rats after myocardial infarction. A**: Survival rate estimated by the Kaplan-Meier method in sham rats (sham; n = 12, none died), MI rats treated by PBS (PBS; n = 12, 7 died), MI rats treated with MSCs (MSCs; n = 12, 6 died), with MSCs-GFP (MSCs-GFP; n = 12, 7 died) and with MSCs-MK (MSCs-MK; n = 12, 3 died). Survival rates of MSCs-MK were significantly higher than for MSCs and MSCs-GFP. **B**: Representative echocardiography findings in the MSCs-MK group before MI (pre-operation) and 4, 8 and 12 weeks after MI (post-treatment). **C**, **D**, **E** and **F**: Measurements were performed at baseline before MI and 4, 8 and 12 weeks after MI, as indicated. **(C)** Left ventricular end-diastolic diameter (LVEDD). **(D)** Left ventricular end-systolic diameter (LVESD). **(E)** Fractional shortening (FS). **(F)** Ejection fraction **(EF)**. *indicates *p< 0.05* vs. the PBS group at the same time-point, one-way ANOVA. All values represent mean ± SEM. MI, Myocardial infarction; MSCs-GFP, Mesenchymal stem cells-green fluorescent protein; MSCs-MK, Mesenchymal stem cells-midkine.

The effect of MK overexpression on the efficiency of cell-based therapy with MSCs was evaluated in a rat model of MI. Two weeks after ligation of the coronary artery, the MI models of rat were confirmed by electrocardiogram, and MSCs, MSCs-GFP and MSCs-MK were injected into the border zone myocardium. We prepared five groups of mice, namely sham and MI plus injection of PBS, MSCs, MSCs-GFP and MSCs-MK. All sham-operated rats survived, whereas only *42%* of the PBS-injected MI mice survived at *12* weeks. Although there was no significant difference in survival rate among the MSCs (*50%*), MSCs-GFP (*42%*) and saline groups, *75%* of rats injected with MSCs-MK survived (Figure 4A).

To evaluate changes in LV dimensions and contractility, echocardiographic analyses were conducted before the surgery and after *4, 8* and *12* weeks (Figure 4B). In the PBS-injected MI rats, a significant increase in LVEDD and LVESD and a significant decrease in fractional shortening (%FS) were observed compared with the sham-operated rats at four weeks and thereafter, suggesting that the LV is dilated and systolic function is compromised. Although both LVEDD and LVESD increased progressively in the MSCs-, MSCs-GFP- and MSCs-MK-injected MI rats (*p< 0.05*) compared with those in sham-operated Rats at *12* weeks (Figure 4C,D), there were no significant differences (*p> 0.05*) among the three groups, suggesting that injection of MSCs, MSCs-GFP and MSCs-MK retard LV dilation but with no difference among them.

The EF and FS in the MSCs and MSCs-GFP groups significantly and progressively decreased from four weeks onwards, suggesting that injection of MSCs and MSCs-GFP prevented LV dysfunction. Furthermore, the FS and EF in the MSCs-MK-injected MI rats gradually decreased, but these values were still significantly higher than other groups (although only EF, *p> 0.05*) (Figure 4E,F). These results suggest that injection of MSCs or MSCs-GFP alone does not have long-term therapeutic effects, but *ex vivo* introduction of MK significantly improves the therapeutic effect of MSCs after MI.

### MSCs-MK survived in long-term follow-up

**Figure 5:**
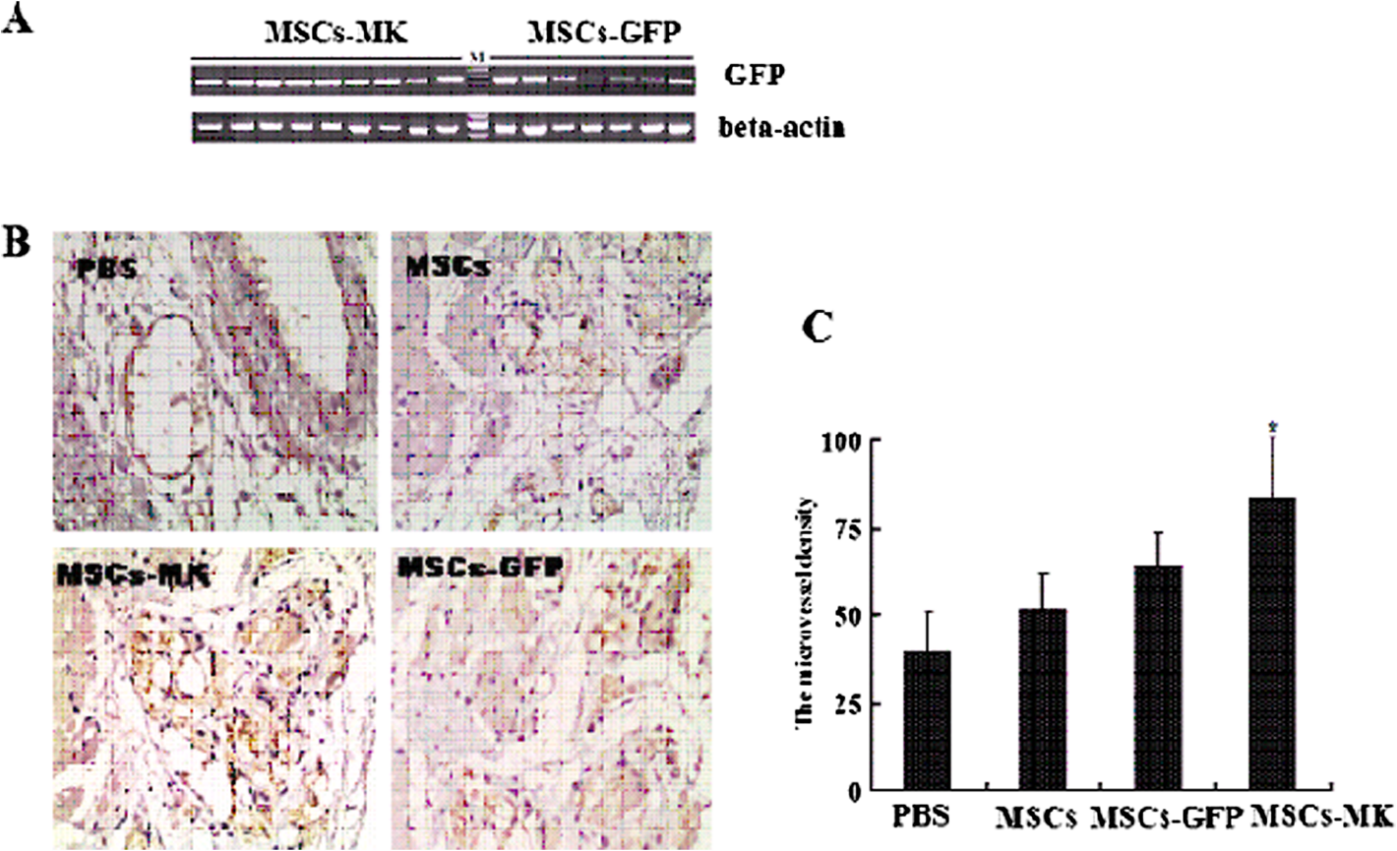
**Histological analysis 12 weeks after therapy. A**: The GFP signal of transplanted cells was determined by PCR **(A)**. **B** and **C**: CD31-positive capillaries in the peri-infarct zone. **C**, Histogram of data shown in **D**. All values represent mean ± SEM. * *p< 0.05* vs. PBS group. The capillary density in the MSCs-MK group was significantly greater than that of the PBS group. GFP, Green fluorescent protein; MSCs-MK, Mesenchymal stem cells-midkine.

After *12* weeks, the survival of transplanted cells was determined by PCR. LV tissue from *10* mice in the MSCs-GFP and MSCs-MK groups was used for detecting the *GFP* gene by PCR. All tissues from animal groups receiving MSCs-MK were more *GFP* positive than MSCs-GFP, while all tissues from the PBS and MSCs animals were *GFP* negative (Figure 5A). The analysis confirmed the presence and survival of the transplanted cells and showed that there were more surviving transplanted cells in the MSCs-MK group than in the MSCs-GFP group.

To detect endothelial cells, the angiogenic effect was determined using immunostaining specific for CD31 expression after the MSC therapy. There was a significant increase in microvessel density in MSCs-GFP *(64.8 ± 8.9,p< 0.05)* and MSCs-MK groups *(83.7 ± 17.2, p< 0.01)* compared with the PBS group *(40.3 ± 10.7),* and not in MSCs group *(51.8 ± 10.3,p> 0.05)*. The microvessel density in the MSCs-MK group was higher than in the MSCs and MSCs-GFP groups (Figure 5B,C).

**Figure 6:**
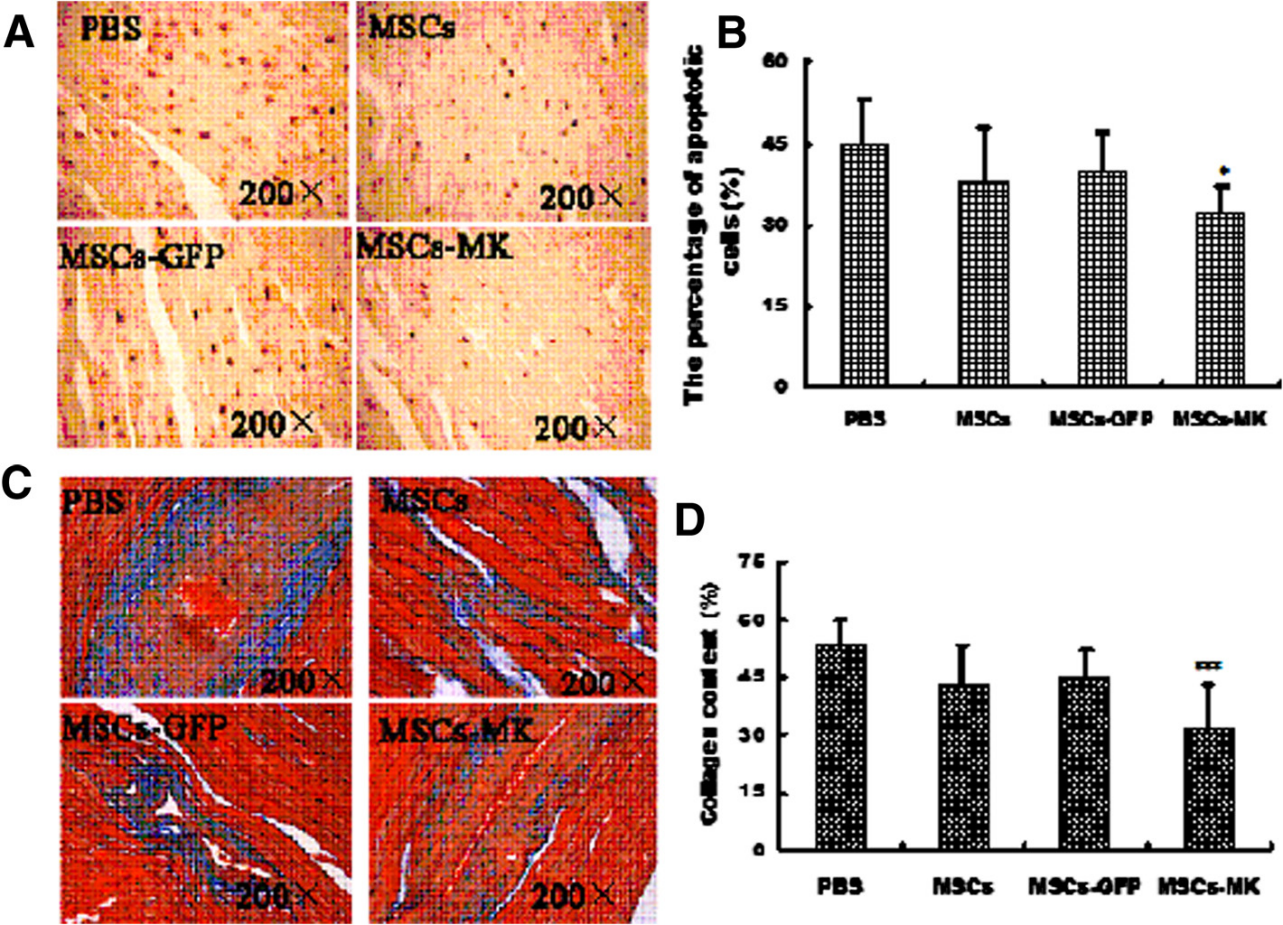
**Representative histology pictures of postmortem apoptosis and fibrosis. A**. and **B**. Apoptosis in the heart tissue as revealed by the TUNEL apoptosis detection kit. **C**. and **D**. Photomicrographs of representative myocardial sections stained with Masson’s trichrome. Myocardium (red) was replaced by fibrous tissue (blue) in all groups in different degrees. Data are shown as means ± SEM. *p< 0.05, ***p< 0.01 vs. PBS group.

In the present study, apoptotic cells were observed using TUNEL stain as a marker. TUNEL-positive cells were recognized in affected myocytic nuclei with chromatin condensation, and were observed in both core and marginal zones. These cells were more common in the PBS, MSCs and MSCs-GFP groups, whereas there were fewer TUNEL-positive cells in the MSCs-MK group and the cells that were present were thinly scattered in the lesion (*p< 0.05*, Figure 6A,B). Masson’s trichrome staining showed extensive myocardial fibrosis in the PBS group. Transplantation of MSCs, MSCs-GFP and MSCs-MK attenuated the development of myocardial fibrosis to different degrees. But only the collagen volume fraction in the MSC-MK groups was significantly smaller than in the PBS group (*p< 0.01*, Figure 6C,D).

## Discussion

Although MSCs are a promising source of cell basic therapy (CBT) for post-MI hearts, MSCs survive poorly when injected into the MI heart, which may limit the functional improvement of the heart through CBT with MSCs [32]. Xing has previously reported that MK promotes self-renewal and proliferation of human embryonic stem cells (hESCs) by inhibiting apoptosis while accelerating the progression toward the S phase and that MK enhances mouse embryonic stem cells (mESCs) self-renewal through the PI3K/Akt signaling pathway [33]. Furthermore, exogenous MK has a protective effect in models of MI of mice and rats. In this present study, we show that *ex vivo* up-regulation of MK in MSCs significantly increases both survival rate and paracrine signaling of the MSCs after injection into the MI heart, thereby significantly enhancing the efficiency of the CBT.

Lentiviruses are promising vectors for delivery of MK transgenes due to their ability to integrate and transduce both dividing and non-dividing cells, which are less constrained by the size of the MK transgene and facilitate its long-term expression [34]. The immunophenotyping analysis in this study revealed that there was no significant difference in the MSC surface markers (positive for CD29, CD44 and CD90 and negative for CD34, CD86 and CD31) before and after the pLenO-DCE vector lentivirus infection that was carried with the GFP + maker, and most MSCs remained stable and undifferentiated after lentivirus infection, suggesting that the infection of MSCs with the pLenO-DCE lentiviral vector did not affect the MSCs and that MSCs infected with the pLenO-DCE lentivirus encoding MK are a promising tool for gene therapy. Thus, our results revealed that a lentivirus titer of 1.5 to 2.0 × 10^9^ TU/ml is promising and adequate for infecting MSCs.

One of the significant challenges in the clinical application of MSCs is the fact that the survival of injected cells is very low *in vivo*, especially in the post-MI heart. Our results show that overexpression of MK in MSCs could prevent the *ex vivo* apoptosis phenomenon induced by oxygen and glucose deprivation, and continued survival of the injected MSCs in the MI heart at 12 weeks was observed only when the MSCs overexpressed MK, suggesting that MK may enhance the survival of MSCs.

The autocrine and paracrine factors whose production is modulated by MK may also be involved in the enhanced survival and protective effects on cardiomyocytes of MK-MSCs. Up-regulation of paracrine factors plays an important role in mediating the beneficial effects of CBT with MSCs. Using qPCR and ELISA analyses, we have identified several growth factors/cytokines, including VEGF, TGF-β and SDF-1a, that are up-regulated in the MSCs-MK cell and its culture substrate. When co-cultured with hypoxic H9C2 cells, overexpression of MK promoted MSCs migration, in part, through the VEGF/VEGFR and SDF-1a/CXCR4 pathways and prevented H9C2 apoptosis by reducing caspase-3 activity. However, these changes are not statistically significant relative to those factors in serum of MI model rats that were treated with MK-MSCs, possibly partly because of individual differences of animals and the factors binding to their receptors.

MK is associated with cancer and serves as both biomarker and therapeutic target. Thus, we have assessed engineered MSC proliferation by CCK8 kits, cell cycle by flow cytometry and telomerase reverse transcriptase (TERT) by Q-PCR. There was no difference among the control cells and MSCs-MK in the cell cycle and the relative expression levels TERT mRNA, but MSCs-MK cells have more potent proliferative capacity than the control MSCs and MSCs-GFP cells. The results showed that MSCs-MK had no tumorigenic effects (Figures S4, S5 and S6 in Additional file 1).

Although injection of either MSCs or MSCs-GFP into the MI hearts slightly but not significantly prevented LV dilation and expansion of MI, it failed to improve LV systolic function, LV remodeling or survival of the animals. However, injection of MSCs-MK remarkably improved these parameters, indicating that MK enhances the efficiency of CBT with MSCs. Because both lentiviral-mediated transduction and stable expression of MK in MSCs improved the efficiency of CBT, the effect of MK is independent of lentiviral transduction. Thus, our results clearly suggest that *ex vivo* modification of signaling molecules, such as MK, can improve the therapeutic potential of MSCs.

### Limitations

The current study has several limitations. First, the functional assay of cord formation by hUVEC was not positive, which may be due to experimental design or test operation. Second, echo evaluation should be performed after MI to avoid potential variability among the animals. On the other hand, we have randomized MI rats into groups after operation, which also eliminated the stress effect to the MI-model rats during the echocardiography detection. Third, the *in vitro* study should be conducted in the neonatal rat cardiomyocytes rather than H9C2 cell line, though this has been validated as a useful model for studying oxidative stress and cardiomyocyte apoptosis. Finally, the *in vivo* imaging detection of GFP fluorescence signals showed that the fluorescence intensity of GFP presented individual difference in groups of MSCs-MK and MSCs-GFP which might be associated with a high degree of model-to-model variability, along with potential variability in sensitivity difference of the detection system.

## Conclusions

In summary, our study suggests that MSCs overexpressing MK transplantation stimulated vasculogenesis effectively via the increase of pro-angiogenesis factors (VEGF, TGF-β), partially prevented hypoxia-induced MSC apoptosis and exerted MSC cytoprotection to anoxia-induced H9C2 cells. For these reasons, this combined strategy of cell transplantation with MK therapy should prove to be a useful approach in the treatment of MI.

## Abbreviations

CBT: cell basic therapy; FCS: fetal calf serum; FGF: fibroblast growth factor; FS: fractional shortening; GFP: Green fluorescent protein; GM-CSF: Granulocyte-macrophage colony-stimulating factor; IGF-1: Insulin-like growth factor 1; LAD: Ligating the left anterior descending coronary artery; LVEDD: Left ventricular end-diastolic diameter; LVEDV and LVESV: Left ventricular end-diastolic and end-systolic volumes; LVEF: The LV ejection fraction; LVESD: LV end-systolic diameter; MI: Myocardial infarction; MK: Midkine; MSCs: Mesenchymal stem cells; OGD: oxygen and glucose deprivation; PBS: phosphate-buffered saline; pLenO-DCE-MK: pLenO-DCE vector encoding MK; SCF: Stem cell factor; SDF-1: Stromal cell-derived factor 1; TGF-β: Transforming growth factor beta; VEGF: Vascular endothelial growth factor.

## Competing interests

The authors have no conflicts of interest to declare.

## Authors’ contributions

SLZ participated in the design of experiments, carried out the molecular analysis of cells, cell transplantation into animals, interpretation and analysis of *in vitro* and *in vivo* data, and helped to draft the manuscript. MHL and XLZ were involved in drafting the manuscript and participated in all experiments involving animals, including histological analyses. YJZ and SLC have been involved in all aspects of the study, including experimental design, analysis and interpretation of data, and manuscript writing. All authors read and approved the final manuscript.

## Supplementary Material

Additional file 1 **This additional file is composed of six figures as follows: Figure S1.** Flow cytometric analysis of MSCs for CD29, CD44, CD90, CD34, CD31 and CD86 expression. A total of 10,000 events were scored. Results are expressed as the percentage of positive cells in the whole population on representative histogram plots. **Figure S2.** Recombinant lentiviral vectors were digested by restriction enzymes. M, DNA maker; 1, pLenO-DCE-MDK; 2, pLenO-DCE. Arrow shows the *MK* gene (421 bp). **Figure S3.** 293 T cells were infected with 0.01 μL pLenO-DEC and pLenO-DCE-MK lentivirus, and the ratio of positive cells were analyzed by flow cytometry. Titer (293 T-transducing units/ml) = 100,000 (target cells) × (% of RFP-positive cells/100)/volume of supernatant (in ml), PLenO-DCE-MK: 1.5 × 10^9^ TU/ml and PLeno-DCE-GFP: 2.0 × 10^9^ TU/ml. **Figure S4.** The Cell Counting Kit-8 (CCK8 kit) was used to monitor cell proliferation. The MSC, MSC-GFP and MSC-MK cells were plated at a density of 5,000 cells/well in 96-well plates. After 24 and 48 hours, 10 μL CCK-8 was added to each well. Absorbance was detected with an enzyme calibrator at 570 nm and then optical density (OD) values were measured. Two batches MSCs-MK cells both have more potent proliferative capacity than the control MSCs and MSCs-GFP cells. **Figure S5.** Analysis of cell cycle distribution was performed by flow cytometry. Representative cell cycle histograms of MSC, MSC-GFP and MSC-MK were shown in this figure. All data of substantial accumulation of cells in G1, G2 and S phase were analyzed, and there was no difference among the control cells and the MSCs-MK. **Figure S6.** Real-time PCR analysis of telomerase reverse transcriptase (TERT) mRNAs, and there was no difference among the control cells and the MSCs-MK. Click here for file
