# Supplementary material for: Mesenchymal stem cells with overexpression of midkine enhance cell survival and attenuate cardiac dysfunction in a rat model of myocardial infarction
Source: Stem Cell Res Ther. 2014 Mar 17;5(2):37. doi: 10.1186/scrt425 (PMC4055147; doi:10.1186/scrt425)
Supplement: Additional file 1 — This additional file is composed of six figures as follows: Figure S1. Flow cytometric analysis of MSCs for CD29, CD44, CD90, CD34, CD31 and CD86 expression. A total of 10,000 events were scored. Results are expressed as the percentage of positive cells in the whole population on representative histogram plots. Figure S2. Recombinant lentiviral vectors were digested by restriction enzymes. M, DNA maker; 1, pLenO-DCE-MDK; 2, pLenO-DCE. Arrow shows the MK gene (421 bp). Figure S3. 293 T cells were infected with 0.01 μL pLenO-DEC and pLenO-DCE-MK lentivirus, and the ratio of positive cells were analyzed by flow cytometry. Titer (293 T-transducing units/ml) = 100,000 (target cells) × (% of RFP-positive cells/100)/volume of supernatant (in ml), PLenO-DCE-MK: 1.5 × 10^9^ TU/ml and PLeno-DCE-GFP: 2.0 × 10^9^ TU/ml. Figure S4. The Cell Counting Kit-8 (CCK8 kit) was used to monitor cell proliferation. The MSC, MSC-GFP and MSC-MK cells were plated at a density of 5,000 cells/well in 96-well plates. After 24 and 48 hours, 10 μL CCK-8 was added to each well. Absorbance was detected with an enzyme calibrator at 570 nm and then optical density (OD) values were measured. Two batches MSCs-MK cells both have more potent proliferative capacity than the control MSCs and MSCs-GFP cells. Figure S5. Analysis of cell cycle distribution was performed by flow cytometry. Representative cell cycle histograms of MSC, MSC-GFP and MSC-MK were shown in this figure. All data of substantial accumulation of cells in G1, G2 and S phase were analyzed, and there was no difference among the control cells and the MSCs-MK. Figure S6. Real-time PCR analysis of telomerase reverse transcriptase (TERT) mRNAs, and there was no difference among the control cells and the MSCs-MK. [file scrt425-S1.doc]

**Additional file 1**


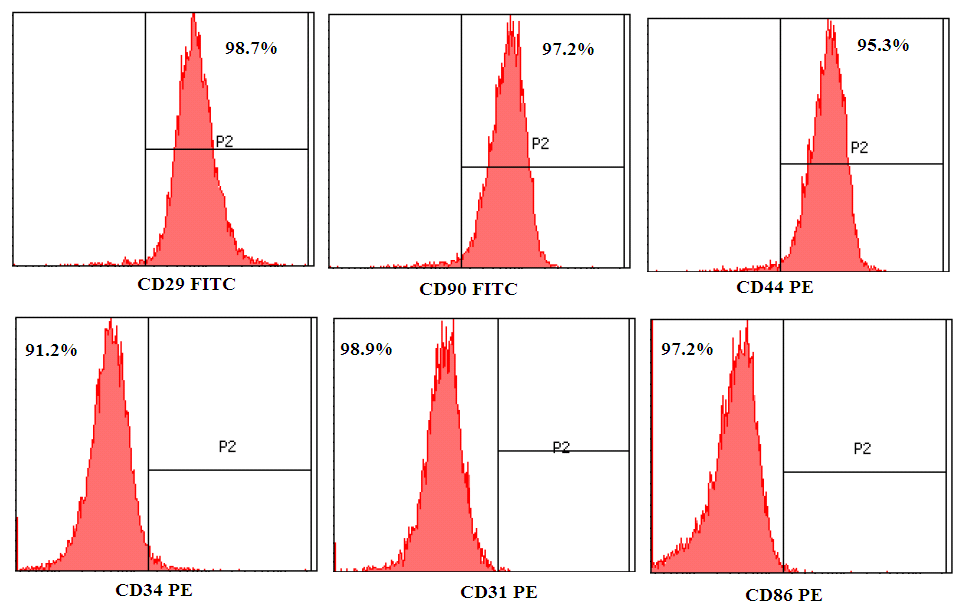


**Figure S1:** Flow cytometric analysis of MSCs for CD29, CD44, CD90, CD34, CD31 and CD86 expression. A total of 10,000 events were scored. Results are expressed as the % of positive cells in the whole population on representative histogram plots.


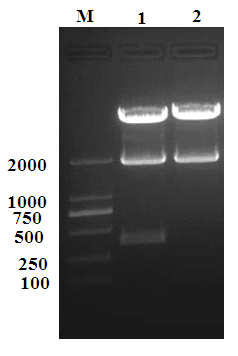


**Figure S2:** Recombinant lentiviral vectors were digested by restriction enzymes. M, DNA maker; 1, pLenO-DCE-MDK; 2, pLenO-DCE. Arrow shows the MK gene (421 bp).


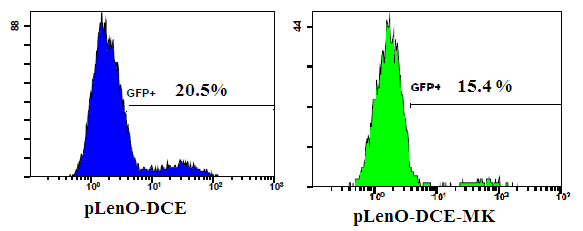


**Figure S3:** 293T cells were infected with 0.01 μL pLenO-DEC and pLenO-DCE-MK lentivirus, and the ratio of positive cells were analyzed by flow cytometry. Titer (293T-transducing units / ml) = 100000 (target cells) x (% of RFP-positive cells/100) / volume of supernatant (in ml), PLenO-DCE-MK: 1.5×10^9^ TU/ml and PLeno-DCE-GFP: 2.0×10^9^ TU/ml.


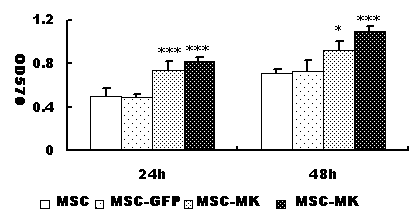


**Figure S4:** The Cell Counting Kit-8 (CCK8 kit) was used to monitor cell proliferation. The MSC, MSC-GFP and MSC-MK cells were plated at a density of 5000 cells/well in 96-well plates. After 24 and 48 hours, 10 µL CCK-8 was added to each well. Absorbance was detected with an enzyme calibrator at 570 nm and then optical density (OD) values were measured. Two batches MSCs-MK cells both have more potent proliferative capacity than the control MSCs and MSCs-GFP cells.


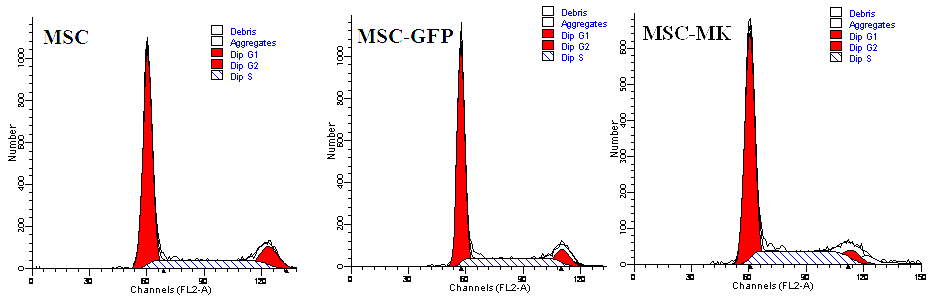


**Figure S5:** Analysis of cell cycle distribution was performed by flow cytometry. Representative cell cycle histograms of MSC, MSC-GFP and MSC-MK were shown in this figure. All data of substantial accumulation of cells in G1, G2 and S phase were analyzed, and there was no difference among the control cells and the MSCs-MK.

**Figure S6:** Real-time PCR analysis of telomerase reverse transcriptase (TERT) mRNAs, and there was no difference among the control cells and the MSCs-MK.
